# Exploring healthcare staff narratives to understand the role of quality improvement methods in innovative practices during COVID-19

**DOI:** 10.1186/s12913-021-07297-0

**Published:** 2021-11-25

**Authors:** Zuneera Khurshid, Eilish McAuliffe, Aoife De Brún

**Affiliations:** School of Nursing, Midwifery and Health Systems, UCD Centre for Interdisciplinary Research, Education, and Innovation in Health Systems (UCD IRIS), University College Dublin, Dublin, Ireland

**Keywords:** Quality Improvement, QI, COVID-19, Healthcare quality, Qualitative research

## Abstract

**Background:**

COVID-19 has impacted the context in which healthcare staff and teams operate and this has implications for quality improvement (QI) work. Contrary to the usual ambivalent relationship staff have with QI work, there have been examples of unprecedented staff engagement in implementing rapid changes during the pandemic indicating a change in important underlying factors that impact staff involvement in QI. The purpose of this study is to explore staff perspectives about how experience and skills of QI methods supported them in implementing innovative practices during COVID-19.

**Methods:**

This is a qualitative narrative study based on narrative interviews to collect healthcare staff stories of implementing rapid change. The stories were identified through social media (Twitter) and a national health magazine issued by the Irish health service. Twenty staff members participated in the interviews. Interviews were audio recorded, transcribed, and anonymised. A four-step thematic analysis was conducted.

**Results:**

The analysis revealed the transformational journey of healthcare staff from the initial shock and anxiety caused by COVID-19 to making sense of the situation, implementing rapid changes, and acknowledging COVID as a learning experience. Six themes were evident from the analysis: COVID anxiety and fear, emotional supports and coping mechanisms, person-centric changes, COVID as a ‘forcing function’ for change, a collective way of working and looking back and thinking ahead.

**Conclusions:**

While most rapid changes during COVID-19 did not represent a systematic and explicit QI application, QI principles were evident throughout the stories and actions taken, including making small changes, testing changes, learning, reflecting as a team, and improving. Many staff members were able to retrospectively identify the relevance of QI principles. COVID-19 eliminated some traditional barriers to change leading to efficient solutions, thus highlighting a need to sustain these positive changes into routine practice to develop an adaptive healthcare system receptive to QI.

**Supplementary Information:**

The online version contains supplementary material available at 10.1186/s12913-021-07297-0.

## Background

### Problem formulation

Healthcare staff want the best for their patients and aspire to improve their services. QI methodology provides staff with a systematic approach to improve their services and outcomes for patients by iteratively testing and measuring change [1]. This seemingly straightforward set of methods is often difficult to translate into action in daily practice. Many staff undertake improvement work regularly without recognising it as such and therefore miss out on the learning from these efforts [2]. Additionally, when healthcare organisations impose demands on their staff to demonstrate engagement in improvement work, staff may see this as an imposition rather than a learning experience [2]. The onset of COVID-19 has impacted the context in which healthcare staff and teams operate as it necessitated rapid changes to maintain safe and effective care delivery. Despite this, the QI mindset, concepts, and tools are still relevant in the current emergency situation and can allow health systems to learn faster, fail better, and learn safely from small scale improvements [3].

Previous research has highlighted an international problem where healthcare professionals are usually reluctant to participate in QI initiatives and this reluctance is more pronounced during periods of organisational turbulence [4]. During the pandemic, the pace of change has been monumental rather than incremental as it required changes in all aspects of care delivery including clinical care, coordination, education, training, performance measurement and reporting. During this time, there has been an outpouring of support for healthcare staff from the public and plethora of stories have been shared in the media and through social media about staff implementing rapid positive changes during the pandemic. Previous studies have often reported mixed outcomes of QI application while some have even reported no statistically significant association of lean methods with patient satisfaction and health outcomes and a negative association with staff satisfaction [5]. However, during COVID-19 staff have reported an increased use and usefulness of QI tools and methods [6]. Previous literature has often highlighted reluctance of healthcare staff to participate in QI implementation, however during COVID-19 staff were actively involved in, and motivated to implement changes. These changes in practice coinciding with the COVID-19 pandemic warranted an investigation of whether healthcare professionals’ skills and experience of QI methods were helpful in implementing innovative practices and to explore other factors supporting effective change during COVID-19.

This rapid change in working context is not without its challenges. A concern raised by studies is the grief, anxiety, uncertainty, and stress experienced by healthcare workers across the globe as they work during the pandemic response [7]. On the other hand, COVID-19 has increased flexibility of health systems by removing bureaucratic processes, enabling rapid changes, multi-disciplinary, national, and international collaborations, and enhanced communication pathways among different actors in the health system [8]. COVID-19 has been a deeply emotional experience for healthcare staff from a personal and professional perspective. Without trivialising these experiences, the pandemic presents a unique opportunity for researchers to document people’s experiences and understand phenomena during a crisis [9]. This also highlighted a gap in research methodologies of QI research being conducted during the pandemic, and the importance of positioning these studies by honouring staff’s overall experience of the pandemic and listening to their voices and stories.

### Purpose or research question

The study aims to identify and collect staff stories of innovative change/improvement practices from the healthcare system during COVID-19 and explore how QI experience and skills supported them in implementing innovative practices during the COVID-19 pandemic. The study also aims to situate these findings in staff’s overall personal, emotional, and professional experience of the pandemic.

## Methods

The assumption of the study is that the rapid changes taking place in the health system have been triggered in response to the pandemic and that staff personal experiences of the pandemic are inseparable from their experiences of implementing rapid changes. This necessitated the use of a research method which accommodates the mixing of personal and professional and provides space to participants to voice their emotions while discussing their experience of rapid change. Therefore, the choice of research design for this study is a qualitative narrative approach. Human beings are naturally inclined towards storytelling as we live storied lives and narrative research gives a voice to research participants to share their experiences and reflections in the form of stories [10]. Additionally, it allows participants the fluidity to move between past, present, and future which is essential for the aims of this research [11]. The study aligns with the constructivist pragmatist paradigm as it relies on participant views to make sense of the phenomenon and acknowledges that participants may make sense of the same phenomenon differently depending on social, and contextual factors [12]. The researcher is familiar with the healthcare system and previously conducted research about the development of QI and measurement skills in healthcare staff during a non-crisis time. The study is reported using Standards for Reporting Qualitative Research (SRQR) guidelines [13].

### Context

COVID-19 is a global challenge and has impacted health systems across the world. Even though research suggests that QI methodologies have played a vital role in rapid implementation of altered care pathways to reduce the transmission of the virus and to ensure high quality care [14]. Health systems struggled to meet the sudden increase in the demand for services without the availability of additional resources. For our research, we are using the stories of change implementation of Irish healthcare staff to understand what motivated them to actively engage in change during this pandemic. The first case of COVID-19 was identified in Ireland on 26th February 2020 and the country went under the first lockdown on 13th March 2020 leading to substantial reorganisation in the health system guided by the need for ensuring safety of staff and service users while maintaining continuity of care. Since then, the Irish health system has withstood three waves of the pandemic, rapidly changing, and adapting. No published studies have addressed this research area using a narrative approach, and the study outcomes and learning will have implications for health systems across the world.

### Sampling strategy

The research team identified stories of QI and change implementation across different settings shared on Twitter and the ‘Health Matters’ magazine of the Irish health system. The search duration was between March 2020–June 2020. All tweets from the head of the Irish health service’s National Press Office’s twitter account, Tweets with hashtag #OurHealthService and stories from the summer edition of the Health Matters magazine were included. The inclusion criteria stated that stories should be related to one or more of the following:

- Healthcare team or healthcare delivered by a team
- Changes in service provision
- Changes to ensure continuous service delivery
- Innovation and initiatives
- Teamwork and leadership
- Quality improvement
- Patient centred care

**Fig. 1:**
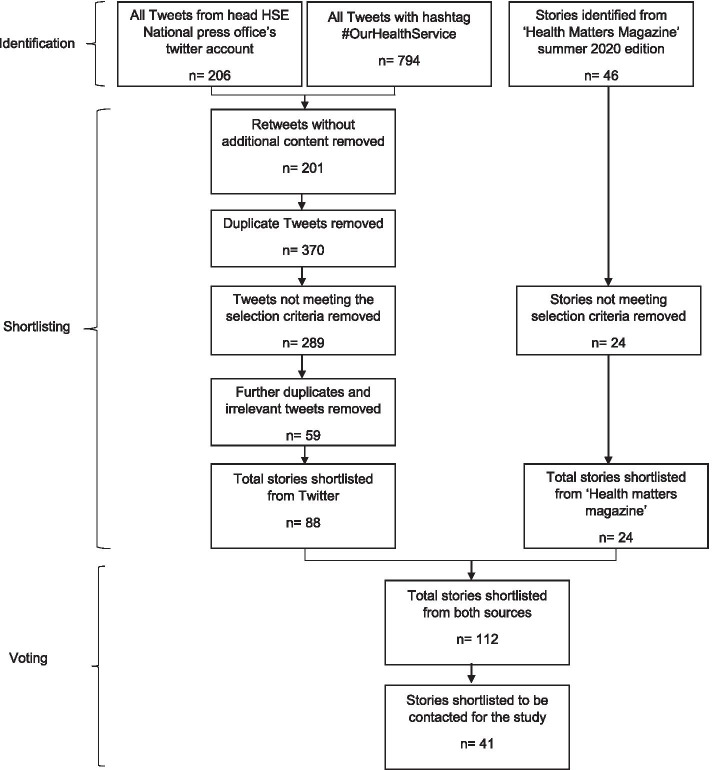
Process flow of story identification and short-listing process

A total of 1048 stories were identified. After a detailed review of stories against the pre-defined criteria and removal of duplicates, 112 stories were selected for further analysis. Next, the research team and representatives from the Irish health service selected the stories that were best exemplars of rapid and innovative changes. Staff from stories selected by two or more people were contacted for participation in the study. Of the 41 stories shortlisted, staff from 19 stories agreed to participate. The study received an exemption from full ethics review from our institution. The process is summarised in Fig. 1.

### Data collection methods

Narrative style interviews were conducted with 20 healthcare staff about 19 stories. A semi-structured interview guide was developed with questions relating to experiences of planning, implementing, and sustaining change during the pandemic. The interviews were structured to be participant led. After the introductory questions, the interviewer requested the participant to narrate their story from beginning to end. The interviewer then asked follow-up questions for clarifications and additional information. Interviews were conducted remotely using phone calls, and videoconferencing programmes including Zoom and MS Teams. Informed consent was obtained from all participants. Interviews were audio recorded transcribed and anonymised. The unit of study is the individual healthcare staff telling their story.

### Data analysis

A qualitative thematic analysis was then conducted for the narrative interviews. Steps of the thematic analysis are presented in Table 1.

**Table 1:** Thematic analysis steps

**Step 1** Thoroughly reading the interview transcripts and making notes of significant emerging issues and developing a summary of the story. Rereading the transcripts to identify attributes of the participants.	
**Step 2** Reading the transcripts to identify initial open codes into an initial table along with a basic definition of the code. When a new code was identified, the researcher re-read previously coded transcripts to re-examine for the new code.	
**Step 3** Grouping the initial codes that are closely related into categories. This process was supported by visual mapping techniques	
**Step 4** Reassessing the categories from step 3 and developing and defining overarching themes	

### Trustworthiness

To increase trustworthiness, a wide range of stories across different settings were collected. For member checking, high level results were shared with 15% (*n* = 3) of the participants. Results were also presented to broader audiences in presentations and webinars and all staff members corroborated the findings.

## Results

Keeping the narrative techniques in mind, the story of each participant is summarised in a chronological manner providing a brief description of past, present, and future. This is presented in Supplemental File 1. Participant attributes are summarised in Table 2.

**Table 2:** Sample characteristics

Characteristic	N
Number of participants	20
Number of stories	19
Pandemic redeployment experience	5
Leadership/supervision/management responsibilities	9
Previous exposure to service improvements*	18
*Examples of service improvements described by participants: Risk assessments, audits, updating policies and QI project completion on topics such as medication safety, length of stay, contamination rates, community access etc)	
**Area of expertise**	
Physiotherapy	4
Nursing	6
Consumer services	1
Occupational therapy	1
QI professional with clinical background	2
Communication	2
Consultant Doctor	1
Physiology	1
Dentistry	1
Podiatry	1

Six themes emerged from the thematic analysis which were COVID anxiety and fear, emotional supports and coping mechanisms, person-centric changes, COVID as a ‘forcing function’ for change, a collective way of working and looking back and thinking ahead.

### COVID anxiety and fear

Pat (clinical nurse specialist) walked into work during the early days of the pandemic engulfed in concern about his own safety, his family’s safety, his patient’s safety, and the loss of life he would have to cope with. He was also aware that this anxiety was a shared experience between healthcare staff and patients:

“Everyone I think was scared in the beginning, who was were going to catch it, how are we going to pass it on to our home, our family, our patients, were they going to be unwell and pass away with us”

Listening to the news at home in March 2020, Clare (Clinical Nurse Education Facilitator) learned about the worsening situation in Italy and Spain and prepared herself to expect the worst. On one hand, the ‘rumour mill’ was churning out unsubstantiated claims which was causing anxiety among staff, David (Doctor) was also concerned about the evolving information and changing guidelines about the virus which was a source of anxiety for him.

Mary (Nurse) was one of the many staff members redeployed into an unfamiliar setting. Her first day of redeployment was like walking into a “war-zone”. For Emma (Clinical Nurse Manager) what made matters worse was that COVID was not impacting a specific aspect of work, it also impacted personal and social life, eliminated simple joys of work life such as having tea with a colleague and dominated all conversations:

"Everyone was absolutely consumed with COVID and talking about how bad it was, what was the next shift going to bring”

Clare (Clinical Nurse Education Facilitator) felt her hands were tied as she could not connect with her patients in the same way and felt the human touch in care slipping away. This was also true for the restricted human interaction between colleagues, staff and their families and patients and their families:

“From a human point of view, if you would have been with people say if bad news is being delivered or something like that, that part is extremely difficult now”

Jake (consultant dentist) found it impossible to plan and felt that he had lost control of his workday. Daniel (physiotherapy manager) described feeling completely overwhelmed and yearned for familiarity and comfort:

“There was numerous days I was going, oh my goodness, I just would much prefer to be back as a therapist, and not have to do this”

### Emotional supports and coping mechanisms

Staff coped in different ways with this anxiety and fear. For David it was a humbling experience (Doctor/microbiologist) and an exercise in admitting his feelings and seeking help when needed. He reflected how this experience has made self-care and looking out for each other a daily practice:

“It was really important to be able to sort of show humility and admit to when I wasn’t feeling okay or when I felt overwhelmed […] concept of self-care and looking out for each other has become much more embedded in what we do on a day-to-day basis”

Mary (Nurse) found comfort and support in her team, talking to them, sharing her fears with them made her realise that they were in this together and the importance of having time for informal social interaction. James (Assistant Director of Nursing) quickly realised that in their enthusiasm to implement rapid changes, his team was at a greater risk of burnout, and he became more proactive in recognising possible signs:

“I suppose there was that kind of hero factor as well, that you know you needed to slow some people down too. You needed to make sure people weren’t burning themselves out”

Jake’s (Consultant Dentist) team felt supported by having access to various training programmes (for example, communication training) through the online training platform in adapting to unfamiliar and stressful situations:

“The thing that some of the team found really helpful, and it was just to kind of keep themselves going on a kind of intellectual level we’ll say, did quite a lot of courses on HSELanD [national online training platform for healthcare staff]”

### Person-centric changes

When the pandemic hit, Lily (Physiotherapist) wondered how she would continue to care for her patients. Her goal was clear, she needed to make changes in the delivery of care processes to ensure quality care for patients while ensuring safety of patients and staff with strict adherence to guidelines:

"Key thing really was that we were optimising patient care during the pandemic and we were trying to work in as safe a way as possible, but while still having a positive impact on our patients"

For James (Assistant Director of Nursing) empathy for the patients, families and staff was the most important element. The optic shifted from only focusing on clinical outcomes to also considering the happiness of staff and patients. He was conscious that patients were lonely and even more vulnerable, and it was up to himself and his team to ensure that they were treated respectfully in their life and in death:

“I was blown away actually by the level of care and compassion that was there you know, seeing the staff do so much for the clients and be there for them and really kind of you know, even for the clients who did pass away the level of compassion. You know, it was such a respectful and it was a humbling experience really”

Staff across the health system such as Alex (Advanced Nurse Practitioner) tried to overcome patient loneliness by arranging video calls with loved ones or Alice (Occupational therapist) printing messages from family and friends and delivering to patients. This engagement with patients and their families to understand their needs led to co-production of services. Jane (QI professional with clinical background) found this co-production and engagement an essential element while developing resources for contact tracers. Alex (Advanced Nurse Practitioner) very humbly explained how she was not doing anything out of the ordinary, COVID-19 has only helped her remember her core duty of care:

“To me it was basic nursing skills, you know, you go in; you talk to the patient, you find out what's important to them and what will make them happy”

### COVID as a “forcing function” for change

Regardless of the geographical locations, our storytellers agreed that COVID acted as a propelling force that accelerated changes that would have taken considerable time to materialise otherwise. George (Podiatrist) had great ambitions for improving his services in the long term and COVID-19 stimulated these changes to fruition:

“I felt like it accelerated my vision of the service. I didn’t think it would happen so quickly but COVID kind of made us do it very fast”

From his days of providing care during the pandemic, Daniel (physiotherapy Manager) remembers being given guidelines in the morning and expected to make the changes by the afternoon. This unprecedented pace of change was a characteristic throughout the pandemic:

“Pre-COVID, about change being slow and incremental, and I suppose staged. Whereas with COVID, it’s been taken out of our hands”

John (Communications officer) remembers spending very little time in planning changes and jumping headfirst into doing and then refining the initiatives on the go. Emma (Clinical Nurse Manager 3) was constantly using formal staff meetings and informal conversations to make and revise small changes quickly. By her third month of redeployment in the national contact tracing programme, Jane (QI professional with clinical background) was expected to be an expert in the new area which required quickly learning and then teaching others. Ann (QI professional with clinical background)) while comparing the usual change and approval process to the process during COVID-19 concluded that the usual barriers to change had been removed:

“We had immediate access to approval, for whatever we were trying to do. In terms of the work we were doing, I mean again, things were done at a far faster pace. Things were approved in an accelerated fashion”

Over the course of delivering services during the pandemic, David (Doctor/Microbiologist) realised that although he had to pause the formal QI programmes in his hospital, the rapid changes taking place in the organisation were an application of QI methods. He also added that in practice, when QI is implemented by staff, it often lacks fidelity to the QI principles and methods which was also true during the pandemic:

“There’s hasn’t been sort of a formal QI programme behind this, in practice a lot of the sort of QI principles have been applied [..] when you actually go and look at the ground level, you discover that people are not following guidelines or policies, they're just doing their own thing.”

On the other hand, Ann (QI professional with clinical background) described the explicit use if QI methodology as the driving force behind the development of the contact management programme:

“From the very beginning, it was very paramount that we were going to adapt, change, try new things, very much along that QI methodology element”

In Pat’s (Clinical Nurse specialist) experience, personal characteristics of staff played a major role in implementing change. He learned that to implement changes, he did not need to get everyone on board and focus on engaged and proactive staff members willing to embrace and drive change. Daniel (Physiotherapy manager) considered himself lucky that he had completed his Masters in change management which helped him in implementing these rapid changes.

### A collective way of working

Looking back, Alice (Occupational therapist) and most of our storytellers experienced an increased sense of togetherness and willingness to support team members. The COVID-experience highlighted the unprecedented potential for resilience and adaptability of teams:

“What it certainly showed for our team was the absolute adaptability and flexibility of the team members. As I said, we had to learn new ways of doing things”

David (Doctor) believed that his team’s pre-COVID dynamics including a flattened hierarchy, resilience, and team decision making helped them adapt quickly to the pandemic. For Jane (QI professional with clinical background) and many others, it was a new experience to see the traditional bureaucracy and siloed ways of working being replaced by a flattened hierarchy:

“There were a lot of things that were a lot easier in that regard. The hierarchy was certainly flattened, a lot of bureaucracy was removed, and that was an amazing way to work”

Being in a leadership role Tom (cardiac physiologist) realised that it is important for him to delegate power and responsibilities during the emergency response for the team to reach its full potential:

“I sort of took a step back and I think again that was another part of allowing people to step into roles and allow my deputy to actually lead that pod and to manage it in the way that she saw fit”.

Sarah (paediatric physiotherapist) reflected on her experience of teamwork and concluded that COVID served as an equaliser, staff from different backgrounds were tossed into a team to achieve a goal in an area they did not have expertise in which helped overcome some of the traditional hierarchal challenges:

“Nobody had done this before, so we were all starting our together. So, it kind of got rid of that hierarchy and that traditional levels of - within the organisation”.

All storytellers cited multidisciplinary teams and working across traditional boundaries as a critical element of COVID response. This created an awareness and recognition in staff about the role of other teams, professionals, and professions. This collective way of working led to the formation of new communication pathways between different departments, teams, organisations, and health systems as well. Lucy (physiotherapist) believed that she had learned to recognise and use the strengths of her colleagues leading to better outcomes for the team. George (podiatrist) along with many of our storytellers described how COVID became a shared goal, making safety the top priority and everything else fell into place:

“What made it easy? Support from our consultants, support from our managers, an enthusiastic team and one single focus. One single goal for the team to focus on”.

### Looking back and thinking ahead

Our storytellers and their teams reported reflecting on how to consolidate the positive changes and mitigate the risks highlighted by COVID. Most storytellers agreed that they intend to retain some or all the changes implemented. Similarly, working from home was a foreign concept for healthcare systems but during COVID, it was crucial in delivering safe care. Those who had the knowledge of change management and conflict resolution found those skills useful in this emergency. There was a great sense of personal and professional achievement among all our storytellers. George (podiatrist) is thinking about how he can collect patient satisfaction data to identify further areas for improvement in his service and remain person centric. Sarah (paediatric physiotherapist) expects an organic spread of this new way of working in the health system:

“The original staff are gone back to their teams, so they’ve taken the learning stuff back you know from the testing service back, to their own teams and said look because this is the way we did it before why can't we try this and we found this really helpful”

However, Jake (consultant dentist) was concerned that some organisations have not moved fast enough to support their staff in adapting to these changes and did not use fit for purpose technologies. Technological challenges were highlighted by most of the storytellers. Organisations should be conscious of patient familiarity and comfort in using technology before pushing ahead with telehealth solutions. Daniel (physiotherapy manager) is trying hard to keep a diary of his reflections about the changes to learn from them, but he is also cognisant of the risk of staff burnout. Tom (Cardiac physiologist) is thinking about tackling the impending burnout and turnover in his team:

“People put so much work in, that will there be a burnout in three or six months, where people just start to leave the service or they go “Do you know what, I’d rather do something else and work in a different environment or go to a private hospital where the working environment is less acute, or different”.

David (Doctor) was circumspective, a health system that is accustomed to a siloed ways of working may quickly snap back to old ways of working:

“There’s still a tendency towards a command-and-control approach both at national level and at hospital group level where it's more that kind of traditional, we are going to just write a policy or a guideline and great it out, tell everybody they have to follow it”

Daniel (physiotherapy manager) aptly summarised this experience as:

“We will look back at this, regardless of what stage of your career, you are and really see that - oh my goodness, it was such a time of challenge, opportunity, and learning”.

## Discussion

The purpose of this study was to explore whether staff skills and experience of QI methods supported them in implementing innovative practices during the COVID-19 pandemic. The stories map the struggles of health service staff in the early phase of the pandemic, how they resolved those challenges and what are their hopes and concerns about the future. These individual and team stories provide an insight into the transformation of the health system during the pandemic by implementing rapid changes. The findings of the research highlight how staff employed coping mechanisms to deal with the stress and anxiety to introduce person-centred and rapid changes. Reflecting on their experiences, staff were appreciative of this new and collective way of working and were contemplating how to best preserve the learning.

Making small changes and gradually refining them is the essence of the QI methodology [15]. Our study showed that the pandemic forced the health system to prioritise and focus on the most important changes rather than trying to take on everything simultaneously. Studies conducted during the pandemic suggest that using proven QI methods, collecting, and regularly reviewing data to inform PDSA (Plan-Do-Study-Act) cycles and rapidly adjusting care pathways not only proved to be useful during the pandemic but can also play a role in reopening services [14]. In this study, few initiatives such as the contact management programme were explicitly driven by the QI methodology and in many sites the ongoing QI activities were halted to focus on emergency response. However, retrospectively, our storytellers were able to see the application and relevance of QI principles in the rapid changes they implemented. Fidelity and authentic application of QI principles is considered important when evaluating success of QI initiatives and many studies have reported finding low levels of QI and PDSA fidelity in practice [16]. During the pandemic, most teams did not explicitly employ authentic QI methods, but relied on implementing and refining solutions based on rapid tests of small-scale change. This raises questions around balancing fidelity against adaptability and flexibility of QI methods depending on the context. This highlights the need for a dialogue between frontline teams and QI educators and healthcare organisations around QI implementation without overburdening staff with documentation.

Rather than starting by helping QI teams understand the need for a specific improvement, many traditional QI approaches focus on the problem and assume that the staff will become committed to the improvement idea on their own [17]. In practice that can lead to staff being less engaged with these initiatives. However, our study revealed a different phenomenon during the pandemic, where everyone across the health system shared the common goal for staff and patient safety. This not only served as a driver for these changes but also a source staff engagement, motivation, and commitment to the changes. Research also suggests that QI teams that include staff from varied disciplines can draw on multiple resources, skills, and collaborations to conquer challenges more efficiently as compared to teams that are restricted to one discipline [18]. Our study confirmed these findings in the pandemic as well as storytellers discussed the important role of working and learning across disciplines, collaborations and benefiting from variable strengths of team members in implementing rapid changes. Healthcare staff are often unenthusiastic about measurement in QI initiatives even though it is a pillar of the QI methodology [19]. During the pandemic there was a different attitude to measurement, teams were self-motivated to collect data from patients and staff to understand the impact of initiatives and to improve them. The goal of measuring change has also expanded to include intangible elements such as patient and staff happiness and morale.

Power, status, and hierarchy can be a barrier to the performance of traditional QI teams [20] however during the pandemic, many staff members were redeployed to unfamiliar situations and new teams were formed that required learning new expertise. The focus was on completing the task and the position in the hierarchy was less relevant as all team members relied on each other for clinical and emotional supports. Studies exploring contextual factors that influence QI initiatives often cite an organisational culture that supports improvement work and the presence of individuals who drive change as important elements [21]. These elements were observed in this study as well. Our storytellers discussed how certain organisations were slow to respond and hesitant to commit resources to facilitate the rapid changes while others with flatter hierarchies found it easier to adapt. The storytellers also talked about the importance of having champions of change who drive the changes on the wards during the pandemic as well. Leadership played a central role during the pandemic. Studies about leadership practices during the pandemic highlight the importance of practicing distributed leadership and concern for staff physical and psychological safety needs [22]. In our study as well, leadership at all levels emerged as an important influence of rapid changes. Leaders willingly shared power with their teams, empowering them to experiment with innovative ideas which spearheaded the implementation of change and improved staff confidence.

Even though there was a need for standardisation and guidelines at the national level, this study highlighted that the traditional command and control approach did not work at an operational level and to implement rapid changes, teams had to apply QI principles. COVID-19 unshackled healthcare staff to deliver bottom-up continuous improvement at the required pace [23]. Instead of ‘slow, staged and incremental’ change, COVID required implementation of rapid changes at an unprecedented pace, forcing staff into a Plan-Do-Study-Act (PDSA) mindset without them realising it. This has made the staff, teams, and organisations more receptive to change however it is uncertain how much of these changed practices will be retained. COVID accelerated the vision of the future for many teams and organisations with the introduction of more efficient solutions and integration into electronic systems but there is also a risk of some organisations and the health system falling back to old, siloed ways of working. Similarly, the immense stress, grief and burnout healthcare staff have endured in their professional and personal lives needs immediate attention. Staff fatigue and burnout has exacerbated during the pandemic, this coupled with a need for rapid team formation, lack of adequate time for patient and self-care can lead to safety and quality problems [24]. The next logical step of individuals, teams, organisations, and health systems should be to prioritise the review of these changes and sustain the ones that work and focus on capacity building among all levels of healthcare staff to make sustainable improvements [7].

The Health Foundation identifies the key elements for effective improvements as visible and focused leadership, effective governance processes, infrastructure and resource availability, skilled and capable staff and a supportive and collaborative culture which encourages learning [25]. All these elements were observed to varying degrees in our study highlighting the global relevance and importance of the study findings. From a methodological point of view, using a narrative approach and collecting stories allowed the staff that they were being listened to and allowed the researchers to understand the stories more intimately. If the researcher focused too narrowly on experience of rapid change and QI without any regard to emotional impact of the pandemic, it would have represented an incomplete story. It also offered staff more flexibility to reach into past events and share their aspirations for the future in relation to the rapid changes that they implemented. A significant step in learning from these stories was to first understand the professional and personal impact of the pandemic through their emotions and feelings which was facilitated by using a narrative approach. Narrative research and storytelling will be an important tool for healthcare leaders and organisations to listen to staff voices and learn from their experiences during the pandemic. Stories of rapid change and QI can also serve as case studies for QI educators as exemplars of bottom-up, staff driven improvements.

### Limitations

The study is based on a limited snapshot of stories collected from one national health system and it might be useful to collect stories from different health systems to compare findings. Additionally, the collected stories are a rich source of information and while this study uses a QI lens to explain the findings, it may be helpful to also analyse the data from other perspectives such as organisational development, leadership, and communication.

## Conclusion

Even in the absence of a systematic approach of QI application, most storytellers discussed making small changes, testing them and iteratively refining solutions which reflects an approximate adaptation of the QI approach without staff members realising it. Many were able to retrospectively identify the relevance of QI principles in the implementation of rapid changes during the pandemic. After the initial shock, grief, and anxiety with the onset of the pandemic, staff employed coping mechanisms and employed a person centric approach to deliver care in an environment which is safe for patients and staff. The pandemic not only accelerated the pace of change but also facilitated it by introducing staff to a new, flexible, and less hierarchical way of working which removed many traditional barriers faced by QI teams. However, to sustain these changes and the improved QI context, there is a need for individuals, teams, organisations, and health systems to reflect, learn and address the risks and challenges that the experience has highlighted.

## Supplementary Information

**Additional file 1: Supplemental File 1.** Summary of stories.

## Data Availability

The datasets generated and/or analysed during the current study are not publicly available due participant privacy/consent conditions who provided consent to use their answers in this research only but are available from the corresponding author on reasonable request.

## Abbreviations

QI: Quality Improvement
COVID-19: Coronavirus Disease 2019
PDSA: Plan-Do-Study-Act
HSE: Health Service Executive
