# Supplementary material for: Exploring healthcare staff narratives to understand the role of quality improvement methods in innovative practices during COVID-19
Source: BMC Health Serv Res. 2021 Nov 25;21:1271. doi: 10.1186/s12913-021-07297-0 (PMC8613456; doi:10.1186/s12913-021-07297-0)
Supplement: Supplementary file 1 — Additional file 1: Supplemental File 1. Summary of stories. [file 12913_2021_7297_MOESM1_ESM.docx]

**Supplemental File 1: Summary of stories**

| **Lily’s story**  I have been working as a physiotherapist in a large university hospital for the past two and a half years. Normally, I work with the Physiotherapy Department in a multidisciplinary team. Together we try to improve our service by applying QI methods. When COVID hit, we had to innovate our physio rehab services while keeping our patients and staff safe, so we decided to utilise the hospital channel that broadcasts Mass for our physio rehab inpatient classes. We also gave our patients supplemental handouts. We broadcasted a 30-minute class daily from Monday to Friday for three months, now we are back to face-to-face sessions. We had great support from the entire hospital although I do wish we had our own camera but despite this, I think our patients benefited from the service. |
| --- |
| **Emma’s story**  I work as a clinical nurse manager in an Emergency Department of an acute hospital. I have 18 years of experience. We have completed many QI projects in our department. We do not have a formal QI team, anyone with an improvement idea can step-up and contribute. Due to COVID, we have split our department into a COVID and non-COVID section. We couldn’t plan ahead much due to the uncertain situation. I observed how COVID had taken over every discussion and there was no fun left in our workday. I wanted to make everyone’s day brighter by reminding them of the good times gone by. I asked colleagues to send pictures of these memories that I could display on a wall so when people see it, they stop and smile. I got a phenomenal response, people sent hundreds of pictures. Everyone got on board and helped me with printing and laminating the photos. And it did make them smile! We all love it, and it is impossible to take the photos off now. I am considering new ways to engage my colleagues and keep spreading this joy. |
| **Lucy’s Story**  I am a respiratory physiotherapist with 20 years of experience and currently working in an acute and community hospital. I am also a team lead. I have only been in this hospital for two years and that has mostly been overshadowed by COVID. My previous service improvement experience proved to be helpful. We divided the team into two pods to minimise chances of spread and had to work 12-hour shifts. We only took on cases for physio that required urgent care following a detailed assessment. Last year we had been able to secure some external funding for setting up a virtual solution to bypass the usual long wait times for pulmonary rehab. This came in handy during COVID as we were able to quickly develop a virtual rehab offering. We found a suitable app to do a one-hour interactive class with our patients. Although our patients miss the social element of coming in for physiotherapy, we had positive feedback. I am not sure whether the rehab will continue face-to-face or virtually or both post COVID. We will decide based on our capacity and patient preferences. |
| **Clare’s Story**  I am a Clinical Nurse Education Facilitator in a children’s hospital where I have been working for the past 30 years. I supervise staff and student nurses and work in the Burns’ unit. I believe we have a great culture of QI in our unit. In my 30 years working here, I have seen the ward evolve but COVID was a completely new experience. Seeing the situation in the rest of the world, I was fearful the same would happen in Ireland. We had to reduce bed capacity, close the playroom and coffee room for parents and reduce the number of staff in the canteen to maintain social distancing. From a human point of view, this has been taxing on all of us especially when working with children, but we were quickly able to adapt our services. We are unsure what the future holds and how long we would have to continue working this way. |
| **Mary’s story**  I am a paediatric nurse with over 30 years of experience. I work in a school for children with complex needs, when COVID hit, the school had to be closed and my colleagues and I were redeployed to a nursing home where most of the staff and patients had contracted COVID. When I found out, I felt scared and didn’t know what to expect. The news only showed the negative side but when we went in, we were relieved to see the preparations in place to keep us safe. It wasn’t easy adapting to the new role. I am a nurse and work with children and now I had to take care of adults. Talking to my family, manager and other colleagues helped me to cope with the stress. I worked there for 5 weeks till their staff returned. Looking back, I think having more information would have eased my anxiety even now, I am unsure if I would be redeployed somewhere again. |
| **Eva’s story**  My name is Eva and I have been working in consumer services of a university hospital for the past 10 years. Over the years I have been involved in various QI initiatives. During COVID, my team and I received many complaints from families not being able to meet patients. This was distressing for the patients. So, we set up an email address where families could send messages, which we would print and deliver to the patients, like our very own post office. We received great support from the management to the frontline. This made patients and families happy and helped staff bond with the patients. Although we don’t need it anymore, but I wonder it may still be useful for patients whose families are in other cities or countries. |
| **Alice’s Story**  I am an occupational therapist specialising in older person care. I have been working in a hospice for the past 6 years during which I have been part of various QI efforts. During COVID, we changed the way we work by taking patients only from hospitals to ensure safety of our staff and patients. Unfortunately, we had to discontinue visitation and to deal with the isolation, we came up with an initiative to connect patients and families virtually. We also created a dedicated email address where families could send messages which we would print and deliver to our residents. We had some tablets donated to us a while back which we were able to use for video calls between patients and families. Although it’s not the same as physical visits, it made patients and families happy. I think this initiative might be useful post-covid for people with families living in other cities. |
| **Sarah’s story**  I am a paediatric physiotherapist and was redeployed to a COVID testing centre. I think COVID was a great equaliser, despite my experience I had to learn from scratch in the testing centre. I had to learn quickly and then teach others. We had to rely on each other to get the testing centre operational. Because of COVID electronic pathways have been set up between GPs and primary care which is a huge win in my perspective, and we will see the benefits post-COVID. Many of the staff have returned to their original roles and have taken the learning back with them. |
| **Alex’s story**  I am currently working as an advanced Nurse Practitioner in a large university hospital group. Normally I work in gastroenterology but during COVID I was redeployed to the patient liaison team. To overcome the isolation patients were facing, we decided to engage with patients and families to find out the things they care about and put it on a board. This board was a great conversation starter for patients and staff. I got a phone and credit to facilitate calls between patients’ families. The patients loved it and I think it helped improve their self-esteem. The visitation rules have been relaxed now so we are not doing this anymore and I have returned to my normal role. |
| **Jane’s story**  I am a speech and language therapist by background and have been working in QI for the past 2 years. COVID has completely changed the way we work and communicate as we are working remotely. When COVID started, I was redeployed to the emergency response to the pandemic. I had little to no information about my role, but I set out to work on designing the contact tracing system along with the other staff. My work focused on developing and refining call scripts. The initial months were exhausting but we were all committed. We are constantly evaluating and improving our work and continuing our ongoing support to the programme |
| **Ann’s story**  I trained and worked as a nurse for many years and have been working in quality improvement for the past 5 years. I was redeployed to the national COVID response to work on developing education resources for contact tracers. Going in, we did not have any defined roles and responsibilities, it was all hands-on deck to get the contact tracing up and running. With time I started to focus more on developing education and training resources for the contact tracers. The entire team supported each other in work and wellbeing. In this rapidly changing environment QI methodology kept us focused on the goal and is also our guide today. |
| **James’s story**  I have been working in substance abuse nursing for the past 25 years and currently work in an inpatient residential treatment. One of the hospitals in our healthcare system was struggling as many of their nursing staff got COVID. They had to employ agency staff to take care of inpatients on a ward with elderly residents. When I noticed this, my team and I volunteered to be deployed. When I went in, there was no leadership or strategy, anxiety among staff was evident and no clear procedures in place. I took on the leadership role, ensured safety of the staff and gave them clear instructions. Once staff felt safe and supported, they went out of their way to deliver care. We became the first unit in that hospital to become COVID free, the patients were happy with the care provided to them and none of the staff got COVID. We have returned to our own unit, but we made sure to do a comprehensive handover paying attention to the likes and dislikes of the person. |
| **John and William’s story**  I am John and I have been working in the communications department in this hospital group for almost 6 years. My colleague’s name is William, and he also works in the same team. Under normal circumstances, we run communication campaigns in our hospital group and serve as the main source of information for staff. We also handle the communication element of QI projects. During COVID, our department came to the forefront in ensuring accurate and timely information is communicated to our staff which was challenging as we were working remotely. We came up with an idea for a staff communication app that could do the job. From planning to implementation, we completed the process within two weeks. We have observed great engagement among the staff with the app and now we are considering ways to keep it relevant in a post-COVID environment. |
| **David’s story**  I am a medical doctor and microbiologist working in a children’s hospital with around 33 years of experience. QI was a part of my role which was pushed to the side because of COVID. I am a team lead and during COVID I had to make rapid decisions and manage the expectations of top management as well. What helped me during this time was the relatively flat hierarchy of my team, which served us well in adapting to the pace of change. I had to actively lookout for my team members to prevent them from experiencing burnout. It gave us the opportunity to work across the traditional silos in our health system and I wonder if we will revert to old hierarchical ways without learning anything from the experience. |
| **Tom’s story**  I am a cardiac physiologist working in an academic teaching hospital. I have almost 23 years of experience. I have participated in various service improvements in the non-invasive cardiology department. During COVID, initially I was unsure whether I would be redeployed to a different setting like many of my colleagues. As events unfolded, we split into pods to ensure safety. The highest priority for us was to devise a way for code permutation emergency cases. We achieved this through collective decision making within our team. Our team has always prized its good team ethos which served us well during the pandemic and we took care of each other. We were also able to digitise our paper-based patient charts which is a huge achievement. As a team lead I took a step back and empowered my team to solve problems which I want to continue in the future. |
| **Pat’s story**  I am a nurse and have 10 years of experience. Currently, I am working in a large university hospital group. I am a ward manager, so I have coordination and supervision responsibilities as well. In 2017, as part of our efforts to become more person-centric, we introduced storyboards for patients on each floor so they could post something that mattered to them such as a picture of their family etc. This was done as a QI project. Now with COVID, patient isolation became very high. We decided to scale this successful idea onto other wards to connect with our patients. It was an immediate success and a great conversation starter, and we hope to continue using it in the future as well. |
| **Daniel’s story**  **I** have been working as a physiotherapy manager for the past 16 months and my role involves supervision of the department. I have completed several QI projects in my past roles. Since COVID, the organisation change process has become integrated with the national change process. The major change my team experienced was the closure of the outpatient department and putting guidelines in place to ensure staff and patient safety. Communication has been a key element. We have relied on physical communication boards on the floors as well as WhatsApp. I just completed a degree focusing on implementing change in health systems which equipped me to deal with this flux. I have been keeping a diary to document all the changes taking place but its hard to keep up. I have also done surveys with the staff to get their feedback about changes. We are exhausted but there has been great learning too and we aim to retain some of the changes such as telehealth. |
| **Jake’s story**  I have been a dentist for the past 20 years and I am currently working in a children’s hospital. I am the lead consultant and I put great value on upskilling my team. Since COVID started, some of my vulnerable staff had to cocoon and we are unable to offer our usual suite of services. Our team tried to implement a few small-scale improvement projects during COVID, but we could not manage it because of our resource constraints which is very frustrating. We had to reduce contact with our patients and our waiting lists are growing which has made our service extremely vulnerable. We have been trying to remain connected and do dental consultations using different communication platforms. We have realised that video consultation is not well suited for children, and we are waiting for an opportunity to return to providing full services. |
| **George’s story**  I have been working as a podiatrist for the past 13 years. I currently work in an acute hospital. Our service is based on national and international models of care for the diabetic foot which all agree that the sooner you see the patient the better. Keeping this in mind, during COVID, we decided to prioritise our most high-risk patients to reduce ED attendance. We would assess and advise patients virtually and refer to other teams such as endocrinology etc depending on the need and only brought in patients if it were unavoidable. Our patients don’t have to come up to the hospital now and it has made our service more integrated with the community. We hope to continue this in future as well. |
